# Tuboovarian Abscess as Primary Presentation for Imperforate Hymen

**DOI:** 10.1155/2014/142039

**Published:** 2014-04-16

**Authors:** Jeh Wen Ho, D. Angstetra, R. Loong, T. Fleming

**Affiliations:** Department of Minimally Invasive Gynaecology, The Gold Coast University Hospital, Level 1B Block North, 1 Hospital Boulevard, Southport, QLD 4215, Australia

## Abstract

*Objective. *Imperforate hymen represents the extreme in the spectrum of hymenal embryological variations. The archetypal presentation in the adolescent patient is that of cyclical abdominopelvic pain in the presence of amenorrhoea. We reported a rare event of imperforate hymen presenting as a cause of tuboovarian abscess (TOA). *Case Study.* A 14-year-old girl presented to the emergency department complaining of severe left iliac fossa pain. It was her first episode of heavy bleeding per vagina, and she had a history of cyclical pelvic pain. She was clinically unwell, and an external genital examination demonstrated a partially perforated hymen. A transabdominal ultrasound showed grossly dilated serpiginous fallopian tubes. The upper part of the vagina was filled with homogeneous echogenic substance. Magnetic resonance imaging (MRI) demonstrated complex right adnexa mass with bilateral pyo-haemato-salpinges, haematometra, and haematocolpos. In theatre, the imperforate hymen was opened via cruciate incision and blood was drained from the vagina. At laparoscopy, dense purulent material was evacuated prior to an incision and drainage of the persistent right TOA. *Conclusion.* Ideally identification of imperforate hymen should occur during neonatal examination to prevent symptomatic presentation. Our case highlights the risks of late recognition resulting in the development of sepsis and TOA.

## 1. Introduction

Imperforate hymen represents the extreme in the spectrum of embryological variations in hymenal configuration, with reported incidence ranging from 0.014% to 0.1% [1]. Antenatal diagnosis of imperforate hymen is challenging; thus, neonatal diagnosis is optimal to prevent symptoms and complications seen when primary presentation occurs in adolescence.

The archetypal presentation in the adolescent patient is that of cyclical abdominopelvic pain in the presence of amenorrhoea [2]. The accumulated blood behind an intact hymen may compress the adjacent pelvic organs or vessels, resulting in some of the less common presentations such as urinary retention, back pain, or constipation. We report a rare case of spontaneous partial rupture of an imperforate hymen resulting in tuboovarian abscess (TOA) and sepsis.

## 2. Case Study

A 14-year-old girl presented to the emergency department of the Gold Coast University Hospital complaining of severe left iliac fossa pain with her first episode of heavy bleeding per vagina since the morning of presentation. Her background history was unremarkable with no significant medical or surgical history. She had no previous sexual encounters (virgo intacta). Further questioning elucidated that she had not gone through menarche and had cyclical pelvic pain for the previous three months.

Examination revealed a clinically unwell patient. She presented as febrile to 39 degrees Celsius, with tachycardia of 118 bpm. Her blood pressure was 120/60, showing a postural drop to 105/40. Her abdomen was soft; however there was marked lower abdominal tenderness on palpation, rebound tenderness and abdominal guarding. External genital examination was limited by copious amounts of both old and fresh blood. However, it did reveal a partially perforated hymen with the rest of the hymen still intact. Intravenous antibiotics were empirically commenced.

Biochemical workup indicated signs of infection, with a c-reactive protein of 315, elevated white cell count of 12, and a neutrophil count of 8.9. Blood cultures were obtained, and ultimately a coagulase negative staphylococcus (probable skin contaminant) was grown. The urine specimen was grossly contaminated with blood and grew no organism. The beta-human chorionic gonadotropin was negative.

**Figure 1:**
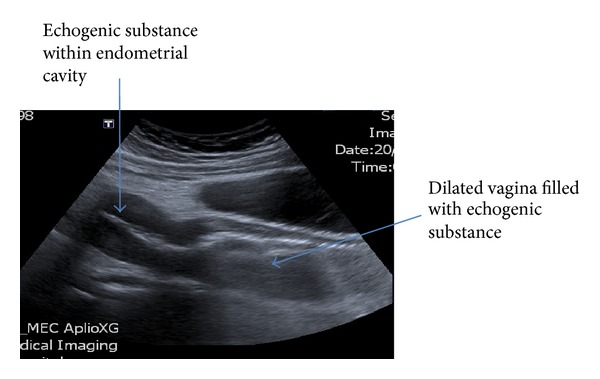
Transabdominal ultrasound of uterus and vagina.

**Figure 2:**
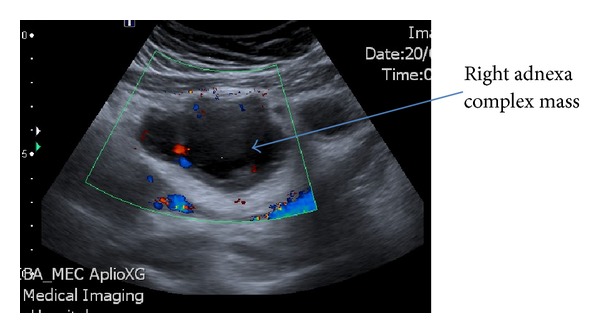
Transabdominal ultrasound of right adnexa-complex mass noted 70 mm × 45 mm × 42 mm.

**Figure 3:**
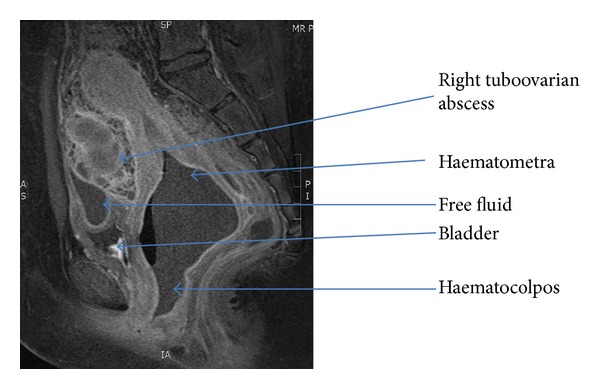
Sagittal view T1 weighted MRI abdomen/pelvis.

**Figure 4:**
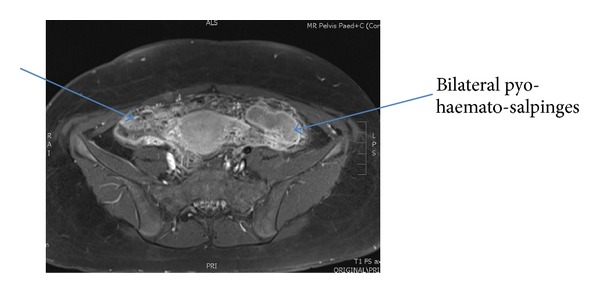
Axial view T1 weighted MRI pelvis.

The transabdominal ultrasound images showed grossly dilated, serpiginous fallopian tubes with low level internal echoes and peripheral vascularity. There was echogenic substance within the endometrial cavity; the upper part of vagina was dilated and filled with homogenous echogenic substance (Figure 1). A right complex mass was noted on ultrasound (Figure 2). The lower vagina was not well visualized. The renal tract was assessed as normal. Magnetic resonance imaging (MRI) further elucidated the complex right adnexa mass with bilateral pyo-haemato-salpinges, haematometra, and haematocolpos (Figures 3 and 4). The pictures were in keeping with a partial imperforate hymen rather than vaginal septum.

**Figure 5:**
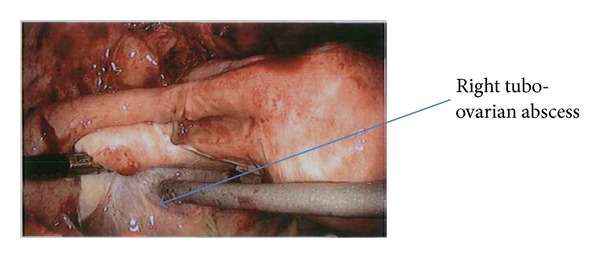
Image from diagnostic laparoscopy procedure.

An examination under anaesthesia with diagnostic laparoscopy was undertaken. The imperforate hymen was opened via a cruciate incision and blood drained from distended and oedematous upper vagina. Purulent material in the abdominal cavity was drained, and division of omental adhesions to uterus and fallopian tubes, caecum, and appendix was necessary to adequately view pelvic organs. A right TOA was identified, incised, and drained (Figure 5). A thorough washout was performed and a pelvic drain was inserted.

She had an uncomplicated recovery and was discharged six days following the procedure with a course of oral antibiotics. At outpatient review six weeks following surgery, she reported a resolution of her dysmenorrhoea with the menstrual cycle experienced postoperatively.

## 3. Discussion 

A literature search was conducted using Medline, Pubmed, and Cochrane with the terms [mullerian anomaly] OR [mullerian tract] OR [imperforate hymen] OR [vaginal septum] AND [pelvic abscess] OR [pelvic infection]. This yielded several case reports of common and unusual presentations for imperforate hymen.

On occasion, antenatal ultrasound can detect an imperforate hymen due to the presence of hydrocolpos in the fetus in response to maternal oestrogens [11]. Imperforate hymen can also be identified on examination of the newborn, and if it is asymptomatic at this time, the recommendation is to delay surgical management until puberty, when the Oestrogenization improves elasticity and healing. The diagnosis is infrequently made in infancy, if the patient presents with a mucocele, in which case hymenectomy may be indicated.

A retrospective cohort study demonstrated a bimodal distribution of age at diagnosis. 43% of patients were diagnosed at less than 4 years of age, and the remaining 57% were diagnosed over 10 years of age. In the group of patients diagnosed over 10 years, 100% of patients were asymptomatic, compared with just 10% of girls diagnosed under the age of 4 years [2].

In spite of recommendations for early inspection of the external genitalia, variations in hymenal anatomy frequently escape diagnosis until menarche. Although the most common adolescent presentation of an imperforate hymen is haematocolpos manifesting as abdominal pain [2], there are several case reports of more unusual or severe presentation of imperforate hymen, and these are presented in Table 1.

Of interest are the two case reports of pelvic infection arising from an imperforate hymen, which demonstrates the breakdown of the barrier from a sterile haematocolpos, either iatrogenically [6], or embryologically [7]. This paralleled our case presentation in that our patient did not become septic until the spontaneous rupture of the hymen which thus facilitated ascending infection of her reproductive tract.

Only one paper reviewed the long term outcome of late diagnoses of imperforate hymen. It reported the persistence of menstrual dysfunction even after hymenectomy, with 60% demonstrating abnormal menstruation and 40% with ongoing dysmenorrhea [11]. Furthermore, the data assessing fertility in these patients is sparse but encouraging. 86% of women who attempted pregnancy conceived after surgical correction of their imperforate hymen [12].

## 4. Conclusion

Ideally identification of the imperforate hymen should occur during the neonatal examination. Delay in diagnosis results in symptomatic presentations, with the potential for long-term menstrual and reproductive ramifications. Our case highlights the risks of late recognition of imperforate hymen, through the development of sepsis and TOA.

## Figures and Tables

**Table 1:** Unusual presentations of imperforate hymen.

Presentation	Authors	Year
Back pain/sciatica	Bapat and Bergsman [3]	2008
	Drakonaki et al. [4]	2010

Recurrent UTI	Bursac et al. [5]	2012

Iatrogenic pyocolpos	Lok and Yip [6]	2001

Pelvic abscess	Sanfilippo and Mansuria [7]	2006

Urinary retention	Dane et al. [8]	2007
	Ercan et al. [9]	2011
	Gyimadu et al. [10]	2009
